# Characterization of Volatile Flavor Compounds in Supercritical Fluid Separated and Identified in Gurum (*Citrulluslanatus* Var. *colocynthoide*) Seed Oil Using HSME and GC–MS

**DOI:** 10.3390/molecules27123905

**Published:** 2022-06-17

**Authors:** Emad Karrar, Isam A. Mohamed Ahmed, Wei Wei, Frederick Sarpong, Charalampos Proestos, Ryszard Amarowicz, Emel Oz, Aly Farag El Sheikha, Ayman Y. Allam, Fatih Oz, Xingguo Wang

**Affiliations:** 1 National Engineering Research Center for Functional Food, Collaborative Innovation Center of Food Safety and Quality Control in Jiangsu Province, School of Food Science and Technology, Jiangnan University, Wuxi 214122, China; emadkarrar26@yahoo.com (E.K.); weiw@jiangnan.edu.cn (W.W.); 2 Department of Food Science and Nutrition, College of Food and Agricultural Sciences, King Saud University, Riyadh 11451, Saudi Arabia; iali@ksu.edu.sa; 3 Value Addition Division, Oil Palm Research Institute-Council for Scientific and Industrial Research (CSIR), Kade P.O. Box 74, Ghana; fsarpong@csir.org.gh; 4 Laboratory of Food Chemistry, Department of Chemistry, School of Sciences, National and Kapodistrian University of Athens, 15772 Athens, Greece; 5 Institute of Animal Reproduction and Food Research, Polish Academy of Sciences, Tuwima Street 10, 10-748 Olsztyn, Poland; r.amarowicz@pan.olsztyn.pl; 6 Department of Food Engineering, Agriculture Faculty, Ataturk University, Erzurum 25240, Türkiye; emel.oz@atauni.edu.tr (E.O.); fatihoz@atauni.edu.tr (F.O.); 7 College of Bioscience and Bioengineering, Jiangxi Agricultural University, 1101 Zhimin Road, Nanchang 330045, China; elsheikha_aly@yahoo.com; 8 School of Nutrition Sciences, Faculty of Health Sciences, University of Ottawa, 25 University Private Ottawa, Ottawa, ON K1N 6N5, Canada; 9 Department of Food Science and Technology, Faculty of Agriculture, Minufiya University, Shibin El Kom 32511, Egypt; ayman_alaam@yahoo.com

**Keywords:** gurum seed oil, supercritical CO_2_ extraction, screw press process, volatile compounds, HS-SPME, GC–MS

## Abstract

In this study, the volatile compound profiles of gurum seed oil were determined using two methods: supercritical CO_2_ extraction (SFE) and the screw press process (SPP). For volatile compounds extraction and identification, headspace solid-phase micro-extraction (HS-SPME) and GC–MS were used, respectively. A total number of 56 volatile compounds were revealed and identified in oil extracted by SFE, while only 40 compounds were detected in extracted oil by SPP. Acids, aldehydes, esters, ketones, furans, and other components were present in the highest ratio in oil extracted by SFE. In contrast, alcohols and alkenes were found in the highest proportion in oil extracted by SPP. In this study, it was observed that SFE showed an increase in the amounts of volatile compounds and favorably impacted the aroma of gurum seed oil. The results reveal that different extraction methods significantly impact the volatile components of gurum seed oil, and this study can help evaluate the quality of the oil extracted from gurum seeds.

## 1. Introduction

With the globally growing demand for vegetable oils, researchers’ primary aim is to explore the utility and functional characteristics of by-products from plant sources. Gurum (Citrulluslanatus var. colocynthoide) is a wild kind of watermelon belonging to the Cucurbitaceae family and is abundantly available in African countries [1,2]. Gurum has historically been utilized in traditional medicine, especially in Sudan, while gurum seeds are plant by-products that are barely used for [1,3]. Gurum seeds play a major role in human nutrition and health due to their high oil content (27–35.5%). Several studies illustrate that gurum seed oil is a rich source of unsaturated fatty acids (UFA), including ω-6, ω-9, and ω-3, which positively impact human health [3,4,5,6,7].

Numerous conventional methods exist for oil extraction. Among these, solvent (SE) or mechanical extraction (pressing) is considered the main system used for commercial and industrial plant oil production [8]. Supercritical fluid extraction (SFE) has several advantages over the traditional method/systems due to its efficiency, eco-friendliness, and greenness [6]. Supercritical CO_2_ is low in surface tension and viscosity, and has a higher diffusion factor that enhances mass transfer [9,10]. Researchers have successfully extracted oils using the SFE method [6,11,12]. Supercritical CO_2_ is used in oil extraction due to the complete dissolving of triacylglycerols (TGA), fatty acids, and cholesterol.

Moreover, CO_2_ is cheap, non-toxic, and has a high purity [6]. In comparison to Soxhlet extraction, the SFE method prevents the problem of pollution of the oil by residual solvents and reduces the cost of production. For yield results, the solvent extraction method is higher than the screw press process and the SFE method. For supercritical CO_2_, pressure and temperature are crucial factors that may impact the oil’s yield. Again, CO_2_ reaches a crucial point under mild (31 °C, 74 bar) conditions in the absence of O_2,_ and it is effective in preserving the bioactive compounds inside [13].

Plant oils are significant to human life owing to their contribution to providing energy, nutritional components, and exhibiting an appealing range of flavors [14,15]. Flavor is a specific parameter and significant quality standard for plant oils. Intuitively, plant oils possess a distinctive aroma [14]. Volatile compounds possess low molecular weights, generating a unique smell, and easily evaporate at room temperature [14]. The flavors of these compounds are believed to be connected with the quantitative and qualitative composition of the volatile components present in the oil [8]. Flavor is considered a significant factor that impactsconsumers’ choices. The flavor of plant oils depends on their diversity, maturity grade, environmental conditions, growing area, storing conditions, and processing techniques [8,16,17].

Processing techniques also significantly impact the concentrations of volatiles and are therefore responsible for causing an alteration in plant oil flavors when different techniques are employed [8,18]. Many research methods have been suggested to isolate and identify the volatile compounds contributing to the distinctive aroma of oils. Among those methods, solid-phase micro-extractions are a simple and fast method for extracting volatile compounds without a solvent. Headspace is a rapid, sensitive technique that can be used without solvents, and is also an economical technique for isolating volatiles’ analytes from the complex matrix. Headspace’s solid-phase micro-extraction gas chromatography technique was used to determine aldehydes in plant oils.

To the best of our knowledge, no published reports mention the volatile compounds of gurum seed oil. Therefore, the present study is the first to provide the profiles of volatile compounds of gurum seed oil. Thus, the aim of this study is to determine the volatile components in extracted gurum seed oil using headspace solid-phase micro-extraction (HS-SPME), later separated and identified using GC–MS (gas chromatography–mass spectrometry). The present study used the oil extracted by supercritical CO_2_ extraction (SFE) and the screw press process (SPP) for comparison purposes.

## 2. Results and Discussion

**Figure 1:**
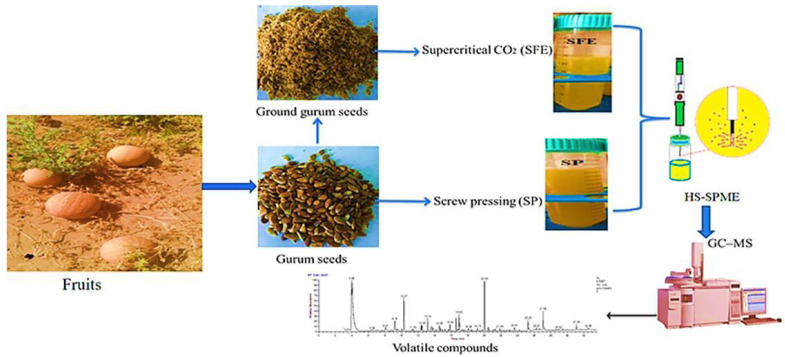
Schematic representation.

The SFE and SPP techniques were used for the oil extraction from gurum seeds. The identification and quantification of volatile compounds were performed using GC–MS, and data from these analyses are depicted in Figure 1.

### 2.1. Acid Compounds

The presence of acids in food is responsible for taste perceptions, such as cheesy and sour notes, which mainly depend on acetic acid (strong, pungent, and sour), octanoic acid (rancid, oily, fatty), and pentanoic acid (cheesy, sweaty, rancid) [18,19,20,21]. In this study, acids were predominantly present in volatile components, accounting for 34.13% of volatile compounds in the oil obtained by SFE, mainly acetic acid (24.81%) and hexanoic acid (5.12%). In comparison, acid components accounted for 12.89% of total volatiles in the oil obtained by SPP, and were mainly hexanoic acid (7.55%), nonionic acid (1.88%), and tetradecanoic acid (1.18%) (Table 1). 

The presence of acetic acid was expected since it is the common volatile compound in most vegetable oil and is formed in the process of the treating the seeds [22,23]. However, acetic acid was present in oil extracted by SFE but not detected in oil obtained by SPP, signifying that extraction methods impacted the concentrations of volatiles, and thus caused an alteration in plant oil flavors. Hexanoic acid, on the other hand, is produced through an enzymatic reaction produced from polyunsaturated fatty acids (PUFA) in oils through the LOX (lipoxygenase) pathway [16,18].

### 2.2. Alcohol Compounds

The presence of alcohol in food products generally produces alcoholic, fruity, sweet, balmy, and green aroma and sensations. However, these aromas depend on the molecular structure of the alcohol produced [21,24]. In this study, alcohols accounted for 6.02% of volatiles in the oil obtained by SFE, mainly endo-borneol (2.71%) and methyl eugenol (1.95%), while alcohol compounds accounted for 15.78% of volatiles in the oil obtained by SPP, mainly 1-hexanol (7.02%), 1-nonanol (1.89%), 2-heptenol (1.29%), and 1-pentanol (1.19%). 

These components played a greater role than other alcohol components in the oil obtained by SPP, especially 1-hexanol. The 1-hexanol compound is an important flavor compound in some vegetables; it produces a green odor, is woody and herbaceous. It is obtained from the bioremediation of UFA (unsaturated fatty acids) and serves as the basis for forming long-chain esters [18,25]. At the same time, endo-borneol and methyl eugenol were found to have the highest ratios in the oil obtained by SFE. 

Borneol elevates the numerous medicinal properties of essential oils in the Dipterocarpaceae family [26]. In traditional Chinese and Japanese medicine, borneol is often used in incense formulas for its uplifting effects on the mind [27]. Methyleugenols are applied as a flavoring in cookies, ice cream, pies, candy, puddings, chewing gums, and cola-based soft drinks [26]. These results demonstrate that different methods of extraction exhibit a significant impact on the flavor components of gurum seed oil.

### 2.3. Aldehyde Compounds

Aldehydes in vegetable oils are fundamentally produced by two pathways: the first is the fat oxygenase pathway (which occurs through the cell fragmentation in the oilseeds), and the second is the automatic oxidation pathway (which occurs in the production and storage process) [18]. In this study, aldehydes were observed to be 12.17% of volatiles in the oil obtained by SFE, mainly 2,4-heptadienal, (E, E) (3.44%), nonanal (2.28%), and 2-undecenal (1.73%), while aldehyde compounds were observed to account for 1.61% of volatile compounds in the oil obtained by SPP, mainly benzaldehyde (0.80%). Nonanal and 2-undecenal are the two main products of linoleic acid oxidation, while 2, 4-heptadienal is the main product of linoleic acid oxidation [18].

### 2.4. Esters Compounds

Ester compounds cover a wider spectrum of flavoring and odor effects; thus, they are widely distributed as the main component in fruit and essential oils. Esters provide a floral, fruity, honey, sweet, and flowery perception in food and other products [21]. In addition to the natural occurrence of ester, the compound can also be produced from the chemical reactions of alcohols and acids [17,28]. Ester components accounted for 9.16% of the total volatiles in oil obtained by SFE and mainly comprised 9(E),11(E)-conjugated linoleic acid, ethyl ester (3.07%), and (E)-9-octadecenoic acid ethyl ester (1.95%), while 9(E),11(E)-conjugated linoleic acid was only found in oil obtained by SPP with a low ratio (0.06%), and it does not have a significantly contribute to the aroma of the oil obtained by SPP (Table 1).

### 2.5. Alkane and Alkene Compounds

Lower amounts of alkene and alkane compounds are always found in oils [23]. In this study, alkane components accounted for 2.76% of the total volatiles in oil obtained by SFE and mainly comprised Dodecanese (0.79%). In comparison, alkene components accounted for 2.21% of the total volatiles, and mainly comprised 3, 5-dimethylcyclopentene (1.34%), squalene (0.60%), and humulene (0.27%) (Table 1). Squalenes are classified as bioactive substances and are known to exhibit beneficial effects on human health [29]; they are also widely available and occur naturally in vegetable oils. Squalenes also act as a precursor to sterols and belong to the terpenoid family [30]. 

In our study, alkane components accounted for 1.66% of the total volatiles in oil obtained by SPP. They were mainly cyclopropane, pentyl (0.84%), and undecane, 2, 6-dimethyl (0.68%), while α-cubebene (2.61%) was the only alkene detected in oil obtained by SPP. This study observed that one terpene (D-Limonene, 0.15%) was also detected in oil obtained by SPP. Terpenes are naturally-occurring compounds in plants and form part of the essential oils [23].

### 2.6. Ketones and Furan Compounds

Ketones are reportedly formed by the β-oxidation of fatty acids (FAs), which produce a few important aromatic compounds [18,31]. In this study, ketone components amounted to 0.79% of the total volatiles in oil obtained by SFE, mainly 2-methyl-6-methyleneoct-7-en-4-one (0.50%), while ketones were not detected in the oil obtained by SPP (Table 1). Various seed oils reported furan compounds [23,31], as previously reported. Furan compounds are either produced by fat oxidation or carbohydrate degradation [23]. In this study, furan components amounted to 0.85% of the total volatiles in oil obtained by SFE, mainly 2(3H)-furanone, dihydro-5-pentyl-(0.54%). In comparison, furan components amounted to 0.78% of the total amount of volatiles in the oil obtained by SPP, mainly furan, 2-pentyl (0.40%).

### 2.7. Other Compounds

Through the other compounds found in oil from gurum seeds, two compounds were classified in this group (Table 1). Vanillin lactoside and l-Gala-l-ido-octose were detected in the oil obtained by SFE, while other compounds were not present in the oil obtained by SPP. Low amounts of vanillin lactoside (0.28%) and l-Gala-l-ido-octose (0.13%) in oil obtained by SFE were also observed. The SFE caused an increase in the amounts of volatile compounds of oils, and this perhaps has a greater impact on the aroma of gurum seed oils.

**Figure 2:**
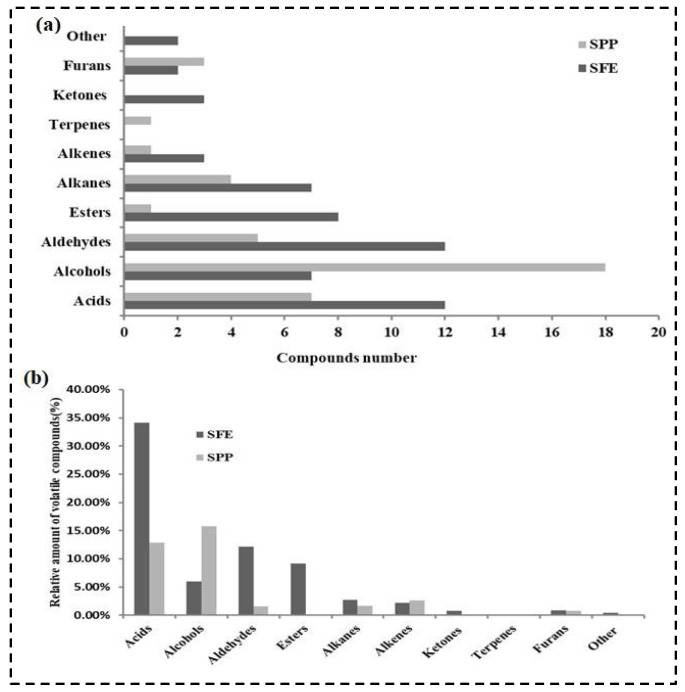
Comparison of volatile components obtained by SPP, SFE and SE of gurum seeds: (**a**) compounds number; (**b**) relative amount of volatile compounds (%).

Volatiles that are studied by humans have a considerably greater effect on the flavor of gurum seed oil. A significant difference was found in the volatile constituent’s oil from gurum seeds obtained by SFE and SPP. For the total volatile compounds studied in this work, a total of 56 volatile components were noted in the oil from gurum seeds by SFE, including 12 acids, 7 alcohols, 12 aldehydes, 8 esters, 7 alkanes, 3 alkenes, 3 ketones and hydroxy ketones, 2 furans, and 2 other compounds. Meanwhile, 40 volatile components were identified in oil from gurum seeds obtained by the SPP, including 7 acids, 18 alcohols, 5 aldehydes, 1 ester, 4 alkanes, 1 alkene, 1 terpene, 1 ketone and hydroxy ketones, and 3 furans, as shown in Figure 2a. 

The results reveal that the oil obtained by SFE could result in the isolation of some characteristic volatile substances. This may explain the great odor differences between the oil obtained by SFE and SPP. Acids, aldehydes, esters, alkanes, ketones, furans, and other components were found to be present in the highest ratios in the oil obtained by SFE compared to SPP (Figure 2b). These results demonstrate that different methods of extraction resulted in a significant impact on the flavor components of gurum seed oil. Alcohols and alkenes were found in the highest yield in oil obtained by SPP compared to SFE (Figure 2b). 

Thus, oils obtained by different methods result in rich bioactive metabolites, ensuring a potential application as an antiproliferative and antioxidant agent in the pharmaceutical and food industries. Furthermore, additional studies on the differences in extraction methods and conditions are required, which could present a clearer view of the metabolic pathways connected with flavor formation.

## 3. Materials and Methods

### 3.1. Raw Materials and Chemicals

Gurum seeds were purchased from Dongola city in Sudan and carried to the National Engineering Research Center at Jiangnan University, China. All chemicals used in the experiments were of high purity. High-purity CO_2_ was employed as a solvent carrier.

### 3.2. Oil Extraction

#### 3.2.1. Supercritical CO_2_ Extraction (SFE)

The oil extracted through the supercritical CO_2_ (SFE) system was obtained through a detailed protocol, as laid out in previous reports [6]. Briefly, gurum (*Citrulluslanatus* var. *Colocynthoideis*) seed oil was extracted by the SFE system equipped with Process Suite software (Waters, Milford, MA, USA) and carefully washed with ethanol. The gurum seed powders were then placed in an extraction vessel and stored for 120 min at 40 °C. The system pressure of 35 MPa was applied with the flow rate of 0.5 L min^−^^1^ of solvent (CO_2_, 100%). The oil was then collected and put into a vacuum drying oven (SLN 75 POL-EKO-APARATURA, Śląski, Poland) under 50 ± 1 °C for 2 h to remove any traces of ethanol. The prepared oils were then stocked at −20 °C until analysis.

#### 3.2.2. Screw Press Process (SPP)

According to the method described in detail in our previous study [6], 1000 g of gurum seeds were used to extract the oil via a screw-press machine. The obtained oil was then stored at 4 °C for further analysis.

### 3.3. Headspace Solid-Phase Micro-Extraction Gas Chromatography–Mass Spectrometry (HS-SPME-GCMS)

The volatile flavor components in gurum seed oil were extracted using headspace solid-phase micro-extraction (HS-SPME), then separated and identified using volatile compounds of gurum seed oil. The gurum seed oil was separated by GC–MS (Scion SQ 456-GC, Bruker, Billerica, MA, USA) using a DBWAX column (30 m × 0.25 mm × 0.25 µm) while maintaining the injector at 250 °C for 3 min. To determine these compounds, 5 g of gurum seed oil obtained by different methods was measured and analyzed following a fully described protocol in our previous report [4]. Each volatile compound profile of gurum seed oil obtained by different extraction methods was separated and identified by comparing mass spectra and RI, as previously demonstrated [4].

### 3.4. Statistical Analysis

Data collection was performed in triplicate and presented as means ± SD through the processing of Origin-Pro 9.2 (Origin Lab Corporation, Northampton, MA, USA). Data comparison was performed using one-way analysis of variance (ANOVA) and Fisher’s multiple comparison tests.

## 4. Conclusions

The present study first reported the characterization of volatile compounds obtained from gurum seed oil by SFE and SPP that were separated and analyzed using GC–MS. A total of 56 volatile compounds were analyzed and identified in gurum seed oil obtained by SFE, compared to 40 compounds obtained by SPP. Acids, aldehydes, esters, alkanes, ketones, furans, and other components were found to be the highest ratio of compounds in the oil obtained by SFE compared to SPP. Alcohols and alkenes had the highest yield in oil obtained by SPP compared to SFE. These results demonstrate that different extraction methods have a significant impact on the volatile flavor composition and overall quality of gurum seed oil.

## Figures and Tables

**Table 1:** Relative content of volatile compounds identified in gurum seeds oil obtained by SFE and SPP using GC–MS.

RT ^a^	Compounds ^b^	Molecular Formula	CAS, No ^c^	Relative Peak Area (%)	
				**SFE ^d^**	**SPP ^e^**
	Acids				
12.35	Acetic acid	C_2_H_4_O_2_	64-19-7	24.81	ND
14.22	Propanoic acid	C_3_H_6_O_2_	79-09-4	0.59	ND
18.05	Pentanoic acid	C_5_H_10_O_2_	109-52-4	0.44	ND
18.06	Pentanoic acid	C_5_H_10_O_2_	109-52-4	ND	0.58
19.99	Hexanoic acid	C_6_H_12_O_2_	142-62-1	5.12	7.55
21.05	8-Phenyloctanoic acid	C_14_H_20_O_2_	26547-51-3	0.10	ND
21.84	Heptanoic acid	C_7_H_14_O_2_	111-14-8	0.55	0.55
23.60	Octanoic acid	C_8_H_16_O_2_	124-07-2	0.80	0.74
25.25	Nonanoic acid	C_9_H_18_O_2_	112-05-0	ND	1.88
25.49	2-Octenoic acid, (E)-	C_8_H_14_O_2_	1871-67-6	0.13	ND
26.05	17-Octadecynoic acid	C_18_H_32_O_2_	34450-18-5	0.13	ND
26.63	n-Decanoic acid	C_10_H_20_O_2_	334-48-5	0.70	0.41
31.09	Tetradecanoic acid	C_14_H_28_O_2_	544-63-8	0.55	1.18
32.64	Octadecanoic acid	C_18_H_36_O_2_	57-11-4	0.21	ND
	Alcohols				
7.91	3-Decyn-2-ol	C_10_H_18_O	69668-93-5	0.07	ND
8.04	1-Pentanol	C_5_H_12_O	71-41-0	ND	1.19
9.57	2-Hexanol, 5-methyl-	C_7_H_16_O	627-59-8	ND	0.47
14.36	Linalool	C_10_H_18_O	78-70-6	0.14	ND
10.27	1-Hexanol	C_6_H_14_O	111-27-3	ND	7.02
12.36	1-Octen-3-ol	C_8_H_16_O	3391-86-4	ND	0.93
12.63	6-Hepten-1-ol, 2-methyl-	C_8_H_16_O	-	ND	0.17
13.60	2-Heptenol	C_7_H_14_O	-	ND	1.29
15.64	2-Octen-1-ol, (E)-	C_8_H_16_O	18409-17-1	ND	0.26
15.74	Ethanol, 2-(2-ethoxyethoxy)-	C_6_H_14_O_3_	111-90-0	0.19	ND
16.56	1-Nonanol	C_9_H_20_O	143-08-8	0.49	1.89
16.69	2-Butyl-2,7-octadien-1-ol	C_12_H_22_O	-	ND	0.19
17.28	endo-Borneol	C_10_H_18_O	507-70-0	2.71	ND
17.58	2-Nonen-1-ol	C_9_H_18_O	22104-79-6	ND	0.20
18.49	1-Undecanol	C_11_H_24_O	112-42-5	ND	0.17
19.47	2-Decen-1-ol	C_10_H_20_O	22104-80-9	ND	0.06
20.46	Benzyl alcohol	C_7_H_8_O	100-51-6	ND	0.57
21.06	Phenylethyl Alcohol	C_8_H_10_O	60-12-8	ND	0.28
22.09	1-Undecanol	C_11_H_24_O	112-42-5	ND	0.20
22.51	2,4-Decadien-1-ol	C_10_H_18_O	14507-02-9	ND	0.05
22.67	Phenol	C_6_H_6_O	108-95-2	0.47	0.18
22.80	Methyleugenol	C_11_H_14_O_2_	93-15-2	1.95	ND
26.27	Ethanol, 2-(dodecyloxy)-	C_14_H_30_O_2_	4536-30-5	ND	0.66
	Aldehydes				
4.66	Hexanal	C_6_H_12_O	66-25-1	0.51	ND
8.99	Octanal	C_8_H_16_O	124-13-0	0.70	ND
9.60	2-Heptenal, (Z)-	C_7_H_12_O	57266-86-1	0.65	ND
11.14	Nonanal	C_9_H_18_O	124-19-6	2.28	0.44
11.86	2-Octenal, (E)-	C_8_H_14_O	2548-87-0	0.57	0.09
13.20	2,4-Heptadienal, (E,E)-	C_7_H_10_O	4313-03-5	3.44	ND
13.35	Decanal	C_10_H_20_O	112-31-2	0.13	0.12
13.77	Benzaldehyde	C_7_H_6_O	100-52-7	0.53	0.80
14.06	trans-2-Nonenal	C_9_H_16_O	-	ND	0.16
14.07	2-Nonenal, (Z)-	C_9_H_16_O	60784-31-8	0.38	ND
18.25	2-Undecenal	C_11_H_20_O	2463-77-6	1.73	ND
19.30	2,4-Decadienal	C_10_H_16_O	2363-88-4	0.87	ND
30.00	cis,cis,cis-7,10,13-Hexadecatrienal	C_16_H_26_O	56797-43-4	0.38	ND
	Esters				
25.95	Pentadecanoic acid, 14-methyl-, methyl ester	C_17_H_34_O_2_	5129-60-2	0.24	ND
26.44	Hexadecanoic acid, ethyl ester	C_18_H_36_O_2_	628-97-7	2.22	ND
28.55	6-Octadecenoic acid, methyl ester, (Z)-	C_19_H_36_O_2_	2777-58-4	0.56	ND
28.71	Pentadecanoic acid, ethyl ester	C_17_H_34_O_2_	41114-00-5	0.43	ND
28.90	(E)-9-Octadecenoic acid ethyl ester	C_20_H_38_O_2_	6114-18-7	1.95	ND
29.02	11,14-Eicosadienoic acid, methyl ester	C_21_H_38_O_2_	2463-02-7	0.23	ND
29.35	9(E),11(E)-Conjugated linoleic acid, ethyl ester	C_20_H_36_O_2_	-	3.07	0.06
29.45	Phthalic acid, hex-3-yl isobutyl ester	C_18_H_26_O_4_	-	0.46	ND
	Alkanes				
7.02	Dodecane	C_12_H_26_	112-40-3	0.79	ND
7.04	Undecane, 2,6-dimethyl-	C_13_H_28_	17301-23-4	ND	0.68
7.71	Hexane, 1,1-diethoxy-	C_10_H_22_O_2_	3658-93-3	0.58	ND
10.26	Cyclopropane, propyl-	C_6_H_12_	2415-72-7	0.40	ND
12.29	Undecane, 4,7-dimethyl-	C_13_H_28_	17301-32-5	ND	0.04
13.43	Dodecane, 2,7,10-trimethyl-	C_15_H_32_	74645-98-0	0.09	0.10
14.55	Cyclopropane, pentyl-	C_8_H_16_	2511-91-3	0.52	0.84
15.45	Hexadecane	C_16_H_34_	544-76-3	0.24	ND
29.12	Dodecane, 2,6,11-trimethyl-	C_15_H_32_	31295-56-4	0.14	ND
	Alkenes				
12.62	3,5-Dimethylcyclopentene	C_7_H_12_	7459-71-4	1.34	ND
13.14	α-Cubebene	C_15_H_24_	17699-14-8	ND	2.61
16.64	Humulene	C_15_H_24_	6753-98-6	0.27	ND
30.95	Squalene	C_30_H_50_	111-02-4	0.60	ND
	Terpenes				
6.89	D-Limonene	C_10_H_16_	5989-27-5	ND	0.15
	Ketones and hydroxyketones				
15.84	Butyrolactone	C_4_H_6_O_2_	96-48-0	0.21	ND
21.11	δ -Dodecalactone	C_12_H_22_O_2_	2305-05-7	0.08	ND
22.03	2-Methyl-6-methyleneoct-7-en-4-one	C_10_H_16_O	19860-68-5	0.50	ND
	Furans				
7.65	Furan, 2-pentyl-	C_9_H_14_O	3777-69-3	ND	0.40
17.26	2(3H)-Furanone, 5-ethyldihydro-	C_6_H_10_O_2_	695-06-7	ND	0.20
22.99	2(3H)-Furanone, dihydro-5-pentyl-	C9H16O_2_	104-61-0	0.54	0.18
32.97	2(3H)-Furanone, 5-dodecyldihydro-	C_16_H_30_O_2_	730-46-1	0.31	ND
	Other compounds				
29.63	Vanillin lactoside	C_20_H_28_O_13_	-	0.28	ND
30.25	l-Gala-l-ido-octose	C_8_H_16_O_8_	-	0.13	ND

All values given are means of two determinations. ^a^ RT, retention time. ^b^ Volatiles identified by the SPME-GC–MS. ^c^ CAS no: Chemical Abstracts Service Registry Number. ^d^ SFE, supercritical CO_2_ extraction. ^e^ SPP, screw press process ND, not detected. ‘-’means there is no CAS no. for this compound.

## Data Availability

Not applicable.
